# Resistome, Virulome, and Clonal Variation in Methicillin-Resistant *Staphylococcus aureus* (MRSA) in Healthy Swine Populations: A Cross-Sectional Study

**DOI:** 10.3390/genes15050532

**Published:** 2024-04-24

**Authors:** Vanessa Silva, Adriana Silva, Raquel Barbero, Mario Romero, Rosa del Campo, Manuela Caniça, Rui Cordeiro, Gilberto Igrejas, Patricia Poeta

**Affiliations:** 1 Associated Laboratory for Green Chemistry (LAQV-REQUIMTE), University NOVA of Lisboa, 2829-516 Caparica, Portugal; 2 Department of Genetics and Biotechnology, University of Trás-os-Montes and Alto Douro (UTAD), 5000-801 Vila Real, Portugal; 3 Functional Genomics and Proteomics Unit, University of Trás-os-Montes and Alto Douro (UTAD), 5000-801 Vila Real, Portugal; 4 Microbiology and Antibiotic Resistance Team (MicroART), Department of Veterinary Sciences, University of Trás-os-Montes and Alto Douro (UTAD), 5000-801 Vila Real, Portugal; 5 Department of Microbiology, University Hospital Ramón y Cajal and IRYCIS, 28034 Madrid, Spainmario.romero2@ues.edu.sv (M.R.); rosacampo@yahoo.com (R.d.C.); 6 Centro de Investigación Biomédica en Red de Enfermedades Infecciosas (CIBERINFEC), Instituto de Salud Carlos III, 28034 Madrid, Spain; 7 National Reference Laboratory of Antibiotic Resistances and Healthcare Associated Infections, Department of Infectious Diseases, National Institute of Health Dr. Ricardo Jorge, 1649-016 Lisbon, Portugal; 8 Centre for the Studies of Animal Science, Institute of Agrarian and Agri-Food Sciences and Technologies, University of Porto, 4051-401 Porto, Portugal; 9 Associate Laboratory for Animal and Veterinary Science (AL4AnimalS), University of Trás-os-Montes and Alto Douro (UTAD), 5000-801 Vila Real, Portugal; 10 Intergados, SA, Av. de Olivença, S/N, 2870-108 Montijo, Portugal; 11 CECAV—Veterinary and Animal Research Centre, University of Trás-os-Montes and Alto Douro (UTAD), 5000-801 Vila Real, Portugal

**Keywords:** MRSA, pigs, antimicrobial resistance, virulence, clonal lineages, WGS

## Abstract

This cross-sectional study investigates the methicillin-resistant *Staphylococcus aureus* (MRSA): its prevalence, antimicrobial resistance, and molecular characteristics in healthy swine populations in central Portugal. A total of 213 samples were collected from pigs on twelve farms, and MRSA prevalence was assessed using selective agar plates and confirmed via molecular methods. Antimicrobial susceptibility testing and whole genome sequencing (WGS) were performed to characterize resistance profiles and genetic determinants. Among the 107 MRSA-positive samples (83.1% prevalence), fattening pigs and breeding sows exhibited notably high carriage rates. The genome of 20 isolates revealed the predominance of the ST398 clonal complex, with diverse *spa* types identified. Antimicrobial susceptibility testing demonstrated resistance to multiple antimicrobial agents, including penicillin, cefoxitin, and tetracycline. WGS analysis identified a diverse array of resistance genes, highlighting the genetic basis of antimicrobial resistance. Moreover, virulence gene profiling revealed the presence of genes associated with pathogenicity. These findings underscore the significant prevalence of MRSA in swine populations and emphasize the need for enhanced surveillance and control measures to mitigate zoonotic transmission risks. Implementation of prudent antimicrobial use practices and targeted intervention strategies is essential to reducing MRSA prevalence and safeguarding public health. Continued research efforts are warranted to elucidate transmission dynamics and virulence potential, ultimately ensuring food safety and public health protection.

## 1. Introduction

*S. aureus*, acting as a commensal bacterium, can also be a facultative pathogen, and the acquisition of antimicrobial resistance in some strains, particularly MRSA, complicates treatment [1]. MRSA poses a significant threat to human health, and its evolution, particularly in the form of livestock-associated MRSA (LA-MRSA), underscores the complexity of interactions between humans and animals [2]. One of the most crucial strains of LA-MRSA is the molecular type ST398, initially found in pigs and later in several other animals and humans, especially those in frequent contact with these animals [3]. MRSA ST398 has increasingly spread worldwide, being prevalent in more than 20 countries, with swine and humans as predominant sources. Indeed, the most prevalent LA-MRSA lineage in North America and Europe belongs to clonal complex (CC) 398 [4]. Analysis of complete genomes has revealed that CC398 isolates separate into discrete phylogenetic categories: one representing an ancestral clade adapted to humans (susceptible to methicillin and tetracycline) and another representing a descendant clade adapted to livestock (resistant to methicillin and tetracycline) [5]. According to Price et al., LA-MRSA could have originated from the human-adapted clade of methicillin-susceptible *S. aureus* (MSSA) CC398. The transition from human to animal hosts likely involved the acquisition of methicillin and tetracycline resistance, along with the loss of phage ΦSa3, which carries the immune evasion cluster (IEC) genes [5]. It has been shown that potential drivers for LA-MRSA ST398 dissemination may include the trading of colonized swine, contaminated transport vehicles, and colonized humans [6]. Pigs have been identified as crucial reservoirs of LA-MRSA over the past two decades, posing a growing threat to global public health [7]. 

The rise in zoonotic diseases due to farmland expansion and climate change has heightened the importance of studying MRSA in swine, especially considering the role of pigs in transmitting important foodborne pathogens [8]. Globally, pork consumption is on the rise, with pork projected to account for a significant increase in meat consumption by 2030 [9]. The increased demand for animal products, including meat, has led to intensive animal production and processing, raising concerns about the transmission of zoonotic diseases to humans through direct contact, indirect environmental exposure, and food consumption [10]. Approximately 60% of human diseases originate from animals, and 75% of new emerging infectious diseases in humans are transmitted from vertebrate animals [11]. Food-producing animals, including pigs, serve as major reservoirs for many foodborne pathogens [12].

In the European Union, the pig sector is economically significant, with pork being the most consumed meat. Portugal, for example, predominantly features large pig farms [13]. The world’s increasing meat production, particularly from pigs, underscores the need for comprehensive studies on antimicrobial-resistant bacteria, especially MRSA, in swine [14]. Therefore, our objective was to investigate the prevalence of MRSA in healthy pigs from various farms across central Portugal, where pig production is more intense. Additionally, we sought to explore antimicrobial resistance, virulence, and clonal lineages through whole-genome sequencing, along with assessing the biofilm formation capacity of MRSA isolates.

## 2. Materials and Methods

### 2.1. Sample Collection

**Figure 1:**
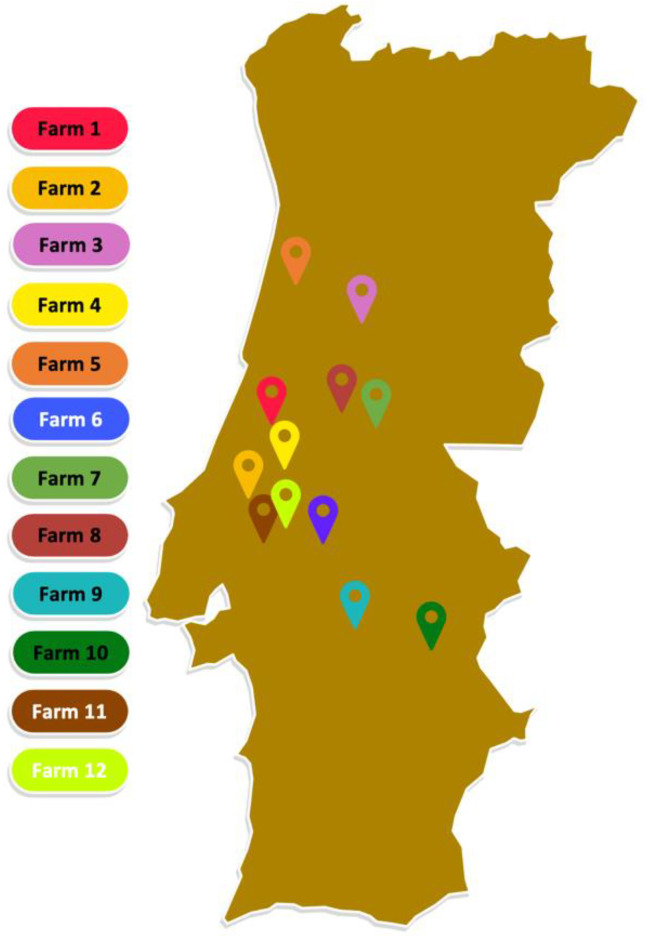
Locations of sampled swine farms across Portugal.

A total of 213 samples were collected from healthy pigs from twelve farms across the center of Portugal from January to October 2021 (Figure 1). Fourteen samples were collected from each farm, with the exception of farm 12, from which only 13 samples were collected. Samples were collected with a nasal and mouth swab (one sample per individual). Of the 12 farms, seven were exclusively breeding pig farms, two were exclusively fattening pig farms, and three were both breeding and fattening farms. In addition, all breeding pig farms included nurseries. Among the 213 pigs sampled, 100 were breeding sows, 60 were fattening pigs, and 53 were piglets. 

### 2.2. MRSA Isolation

The swabs were placed into tubes containing 5 mL of Brain Heart Infusion (BHI) broth (LiofilChem, Via Scozia, Livorno, Italy) supplemented with 6.5% NaCl and incubated at 37 °C for 24 h. Subsequently, the inoculum was applied to CHROMagar MRSA (CHROMagar, Paris, France) plates to facilitate the isolation of MRSA. Up to three colonies displaying *S. aureus* characteristics but exhibiting morphological variations were collected from each plate. Identification of the *S. aureus* species was conducted through biochemical tests (catalase, DNase, and coagulase) and Bruker Biotyper MALDI-TOF MS (Bruker Daltonics, Bremen, Germany).

### 2.3. Antimicrobial Susceptibility Testing

Antibiotic susceptibility testing was conducted on all isolates, and their susceptibility profiles were determined using the Kirby–Bauer disk diffusion method. Fourteen antimicrobial agents were employed for testing, including (concentration/disk) chloramphenicol (30 μg), tetracycline (30 μg), clindamycin (2 μg), cefoxitin (30 μg), penicillin (1U), erythromycin (15 μg), kanamycin (30 μg), mupirocin (200 μg), gentamicin (10 μg), tobramycin (10 μg), linezolid (10 μg), ciprofloxacin (5 μg), trimethoprim–sulfamethoxazole (1.25/23.75 μg), and fusidic acid (10 μg). The determination and interpretation of results adhered to the standards set by the European Committee on Antimicrobial Susceptibility Testing (EUCAST, 2018), with the exception of kanamycin, for which the Clinical and Laboratory Standards Institute guidelines (CLSI, 2017) were followed. *S. aureus* strain ATCC 25,923 served as the quality control in all assays.

### 2.4. Pulsed-Field Gel Electrophoresis (PFGE)

In order to select unrelated strains of MRSA, DNA from all isolates was extracted following the protocol of Ruiz-Barba et al. To perform PFGE, SmaI-digested fragments were separated using electrophoresis under the conditions of 5 to 15 s for 10 h and 15 to 60 s for 13 h. A dendrogram analysis of PFGE profiles was conducted using the UPGMA method based on the Dice similarity index, with data analyzed using INFO QUEST software version 5.1 from BioLine.

### 2.5. Whole Genome Sequencing (WGS)

In accordance with the results obtained from the PFGE, 20 MRSA isolates were selected to be further studied by WGS. A comprehensive whole genome analysis was conducted using the TORMES v1.3.0 bioinformatics pipeline. This involved the implementation of quality filtering for raw reads (Prinseq version 0.20.4), assembly (SPAdes version 3.14), and subsequent quality assessment (QUAST version 5.1). Various functionalities, including taxonomic identification (Kraken2, RDP Classifier, Barrnap) and annotation (Prokka, Prodigal), were encompassed by the pipeline. Additionally, specific programs for typing (MLST, fimHTyper, and SerotypeFinder), identifying resistance genes (Blastn, ABRicate, and PointFinder using ResFinder, CARD, ARG-ANNOT, and PointFinderDB databases), and exploring virulence factors (Blastn, ABRicate, and VFDB) were integrated. Lastly, the generation of the report and accompanying plots is facilitated by the utilization of R packages, including ggplot2, ggtree, knitr, plotly, RColorBrewer, reshape2, and rmarkdown. Phylotyping was undertaken using ClermonTyper, accessible online at http://clermontyping.iame-research.center/ (accessed on 1 August 2023).

## 3. Results

### 3.1. Prevalence and Phenotypic Antimicrobial Resistance

**Figure 2:**
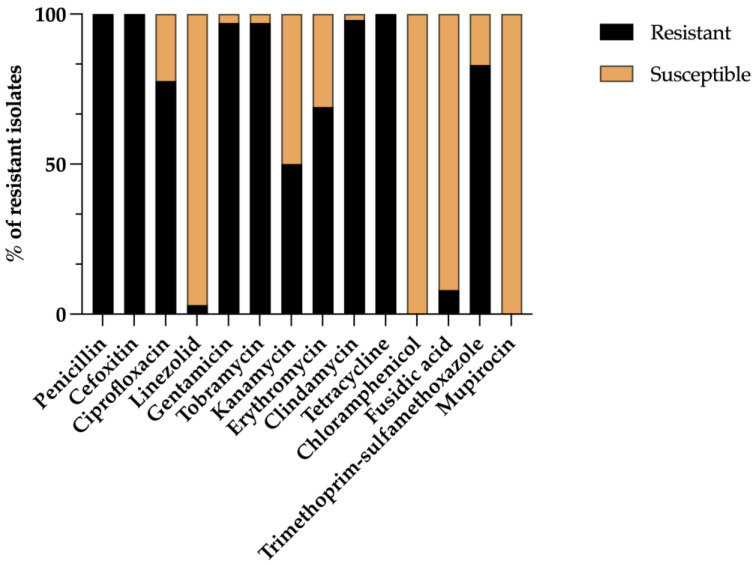
Phenotypic resistance profile of 107 MRSA isolates.

A total of 213 animals were sampled, of which 167 were positive for MRSA, representing 78.4% of positive samples. To confirm the methicillin resistance, all isolates were initially screened for the *mec*A gene, which was present in all strains. It is noteworthy that out of the 53 sampled piglets, 21 (39.6%) were found to be carriers of MRSA. Similarly, among the 60 sampled fattening pigs, 51 (85.0%) were positive for MRSA, while among the 100 sampled breeding sows, a significant majority of 95 (95%) were identified as MRSA carriers. MRSA strains were isolated from the following farms: 16 from farm 1, 15 from farm 2, 12 from farm 3, 11 from farm 4, 18 from farm 5, 15 from farm 6, 12 from farm 7, 12 from farm 8, 14 from farm 9, 9 from farm 10, 18 from farm 11, and 15 from farm 12. The antimicrobial susceptibility was evaluated in all isolates against 14 antibiotics, and the results are shown in Figure 2. As expected, all isolates were resistant to penicillin, cefoxitin, and tetracycline, as well as to clindamycin. Significantly high rates of resistance were noted for gentamicin (97.2%), tobramycin (97.2%), trimethoprim–sulfamethoxazole (83.3%), ciprofloxacin (77.7%), and erythromycin (69.4%) among the isolates tested. Out of the 177 isolates, only five (2.8%) exhibited resistance to linezolid, while 15 (8.3%) isolates showed resistance to fusidic acid. None of the isolates showed resistance to chloramphenicol or high-level mupirocin. Finally, all isolates were classified as multidrug-resistant as they were resistant to at least three antimicrobial classes. 

### 3.2. Whole Genome Sequencing

After performing PFGE and analyzing their clonality by the band pattern, 20 isolates were selected for WGS. The genome assembly was characterized by an average sequencing depth of 716x, with the number of contigs ranging from 92 to 1606 and genome sizes between 2,813,677 and 5,638,892 nucleotides. The average GC content across the samples was 50.65%. The complete draft genome sequences of the 20 MRSA isolates analyzed in this study have been deposited in the National Center for Biotechnology Information (NCBI) GenBank database under BioProject number PRJNA1006036. The individual accession numbers for these sequences range from SAMN37007390 to SAMN37007370. 

**Figure 3:**
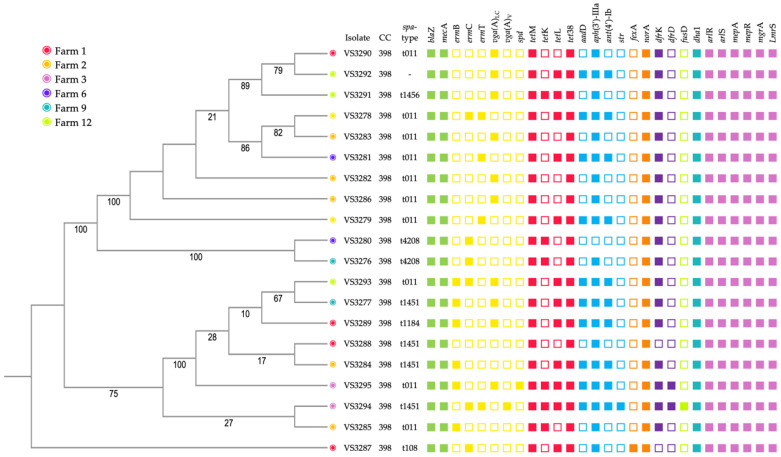
Phylogenetic tree of 20 MRSA isolates subjected to WGS, displaying MLST, *spa*-type, origin farm, and resistance genes.

A pangenome analysis of the 20 MRSA genomes identified 4 core genes, 7833 shell genes, and 14,552 cloud genes. This analysis allows us to explore the genetic diversity within the population. The core genome dendrogram visually depicts the genetic distances between the isolates (Figure 3). Interestingly, the dendrogram reveals significant genetic divergence between MRSA strains VS3290, VS3289, and VS3287, despite their isolation from the same farm. This observation is evident from the substantial differences in the branch lengths within the dendrogram.

### 3.3. Antimicrobial Resistance and Virulence Genes

Among the 20 isolates studied by WGS, all carried the *bla*Z, *mec*A, and *dha*1 genes, which confer resistance to penicillin and cephalosporins. All isolates displayed resistance to macrolides and lincosamides. The most prevalent resistance gene was *vga*(A)_LC_ (*n* = 10), followed by *erm*B and *erm*C (*n* = 6 each). Other detected resistance genes included *erm*T (*n* = 4), *vga*(A)_V_ (*n* = 1), and *spd* (*n* = 1). All isolates carried both the *tet*M and *tet*(38) genes, conferring resistance to tetracycline. Additionally, 12 isolates harbored the *tet*L gene, and 5 isolates had the *tet*K gene, further contributing to tetracycline resistance. All analyzed isolates displayed aminoglycoside resistance, with *aph*(3′)-IIIa present in all but one. Additionally, 10 isolates harbored *ant*(4′)-Ib, 10 isolates carried *aad*D, and one isolate contained the *str* gene. All 20 selected isolates harbored the *nor*A gene, conferring resistance to quinolones. Additionally, one isolate possessed the *fex*A gene, potentially contributing to further resistance. Regarding resistance to trimethoprim–sulfamethoxazole, 18 out of the 20 isolates were found to carry the *dfr*K gene, while two isolates additionally harbored the *dfr*D gene. Finally, one isolate carried the *fus*D gene, which confers resistance to fusidic acid. All isolates harbored genes encoding several multidrug efflux pumps, including *arl*R, *arl*S, *mep*A, *mep*R, *mgr*A, and *Lmr*S.

Analysis of virulence factors identified 49 associated genes. Notably, 18 out of the 20 isolates harbored all 49 genes. However, isolates VS3289 and VS3291 lacked the *spa* gene, which encodes a protein precursor for immunoglobulin G (IgG)-binding protein A. Among the identified virulence genes were those encoding for adenosine synthase A (*ads*A), capsular polysaccharide synthesis enzymes (*cap*8A, *cap*8B, *cap*8C, *cap*8D, *cap*8E, *cap*8F, *cap*8G, *cap*8L, *cap*8M, *cap*8N, *cap*8O and *cap*8P), clumping factor A fibrinogen-binding protein (*clf*A), *Staphylococcus* coagulase precursor (*coa*), cell surface elastin binding protein (*ebp*), type VII secretion system proteins (*esa*A, *esa*B, *ess*A, *ess*B and *esx*A), glycerol ester hydrolase (*geh*), hemolysins (*hlb*, *hld*, *hlg*A, *hlg*B, *hlg*C and *hly*/*hla*), hyaluronate lyase precursor (*hys*A), proteins involved in polysaccharide intercellular adhesin (PIA) synthesis (*ica*A, *ica*B, *ica*C, *ica*D and *ica*R), iron-regulated surface determinant proteins (*isd*A, *isd*B, *isd*C, *isd*D, *isd*E, *isd*F and *isd*G), triacylglycerol lipase precursor (*lip*), I Immunoglobulin G binding protein (*sbi* and *spa*), fibrinogen-binding bone sialoprotein-binding protein (*sdr*E), NPQTN specific sortase B (*srt*B), and staphylococcal surface proteins (*ssp*A, *ssp*B and *ssp*C) (Figure 3).

### 3.4. Molecular Typing

The sequence type (ST) along with the clonal complex (CC) were identified in all isolates. All strains belonged to ST398, which is grouped in CC398. *spa*-typing revealed diversity within the isolates, with six distinct *spa* types identified: t011 (*n* = 10), t1451 (*n* = 4), t4208 (*n* = 2), t1456, t1184, and t108. Notably, strain VS3292 harbored a unique, yet to be officially curated, *spa* type.

## 4. Discussion

This study’s findings highlight a significant prevalence of MRSA among different groups of pigs, with a total of 83.1% of the samples testing positive for MRSA. Specifically, the MRSA prevalence rates varied across different pig groups, with piglets showing a 39.6% carriage rate, fattening pigs at 85%, and breeding sows at a notably high rate of 95%. These findings are consistent with other research indicating that livestock, particularly pigs, are a significant reservoir for MRSA, which can potentially spread to humans, posing public health risks [3,15]. Furthermore, the high prevalence of MRSA in pigs at different stages of production (piglets, fattening pigs, and breeding sows) suggests that these animals may serve as a continuous source of MRSA contamination within the swine industry. MRSA prevalence rates in pigs demonstrate significant variation across different pig groups, indicating a complex epidemiological pattern that is influenced by various factors such as age, management practices, and possibly genetic predisposition. Moreover, piglets exhibited an MRSA carriage rate considerably lower compared to fattening pigs and breeding sows, suggesting that the prevalence of MRSA increases as pigs grow older or as they are exposed to environments with higher MRSA contamination. Nevertheless, in a recent study conducted in Slovenia at two separate pig farms, the prevalence of MRSA in piglets varied significantly, reaching 87.5% in one farm and 33.3% in the other [16]. The high prevalence of MRSA in breeding sows could also be linked to the use of antibiotics such as tetracycline or zinc in animal feed, which have been shown to increase the number of ST398 bacterial cells present in the pigs’ nostrils but have no impact on the transmission of MRSA [17]. The prevalence of MRSA in pigs in Europe varies significantly by country, reflecting diverse agricultural practices, surveillance efforts, and antimicrobial usage policies across the continent [18]. The European Food Safety Authority (EFSA) reported that prevalence rates of MRSA in fattening pigs varied from 12.5% in Slovakia (2022) to 53.6% in Switzerland (2021), with the highest recorded prevalence being 80% in Belgium (2022). Additionally, in 2022, Belgium reported MRSA prevalence in 45.3% of breeding pig herds. Conversely, MRSA was not detected in any pig herds in Norway in both 2021 and 2022 [19]. Although MRSA prevalence in Belgium was similar to that obtained in our study, it was notably lower in other European countries. Indeed, Portugal appears to have a significantly higher MRSA prevalence in pigs compared to the European average. In a study conducted in Portugal in 2017 involving two farms, it was found that 99% of the sampled pigs were colonized by MRSA [20]. This prevalence rate appears to have remained unchanged over time. A recent study conducted in pig abattoirs in Portugal revealed that 98.8% of the samples tested positive for MRSA, indicating an even higher frequency than observed in our own study [21]. Therefore, given that MRSA-colonized pigs pose a threat to public health, there is an urgent need for continued monitoring of MRSA in pigs, particularly in countries with high prevalence rates.

In contrast, a study conducted in the Tohoku region of Japan, which included pigs imported from Europe and North America, found that 32.8% of the pigs were positive for MRSA, with ST398 MRSA isolates obtained [22]. This suggests that the prevalence of MRSA in pigs can also be influenced by international trade and the movement of livestock. In our study, all MRSA isolates were ST398 (CC398). In the EFSA 2023 report, CC398 was the sole clonal complex identified in swine populations during both the years 2021 and 2022. Since its emergence in 2005, MRSA ST398 has been spreading throughout Europe, often associated with distinct *spa*-types, such as t899, t108, t034, t2346, t011, t567, and t1197, with *spa*-type t011 being the most commonly encountered [23,24]. In our study, *spa*-type t011 was also the most frequent, followed by t1451 and t4208. These results are consistent with those obtained in a recent study on pigs from Spain [25]. However, in the study by Leão et al., conducted with pigs sampled in slaughterhouses in Portugal, *spa*-type t011 was the most common, followed by t108 [21]. Another study from Portugal involving pigs in slaughterhouses found that the only *spa* types detected among CC398 MRSA strains were t011, t108, and t1451 [26]. In our study, only one isolate belonged to *spa*-type t108. The other *spa* types found in the study seem to also be related to CC398 [27,28]. Since its initial characterization as LA-MRSA CC398 in 2005 [29,30], this clonal complex has demonstrated a notable capacity to endure within the European swine industry. Studies have indicated a notable correlation between the presence of a specific single-nucleotide polymorphism on chromosome 12 in pigs and the colonization of MRSA in the nasal passages, potentially contributing to the resilience of MRSA in swine production [31]. The ST398 MRSA strain, frequently linked with livestock, has been recognized as a prevalent lineage in pigs and has demonstrated zoonotic transmission potential to humans, particularly individuals in direct contact with these animals [29,30]. Livestock are widely recognized as the primary reservoir of MRSA CC398. However, this lineage exhibits a dichotomy, comprising the classical LA clade and the human clade [31]. It is hypothesized that ST398 initially originated as a clone associated with humans before adapting to animals through the acquisition of tetracycline resistance alongside the loss of integrase group 3 prophages containing the immune evasion cluster (IEC) system genes [32,33]. Moreover, CC398 isolates display antimicrobial resistance patterns similar to those among them, particularly resistance to tetracycline, which is frequently observed and linked with livestock [34].

In accordance, all isolates in our study were resistant to tetracycline and carried *tet*M, *tet*L, *tet*K, and/or *tet*38. The genes *tet*M and *tet*38 were present in all isolates. Resistance to tetracycline can manifest through two primary mechanisms: active transportation facilitated by efflux pumps (encoded by *tet*K and *tet*L genes) and protection of ribosomes conferred by *tet*M and *tet*O genes [35]. *tet*38 is highly conserved in *S. aureus* strains and chromosomally encodes for an efflux pump [36]. The tet38 efflux pump is regulated by *mgr*A, which is an indirect negative regulator of *tet*38 gene expression [37]. However, all our isolates also carried the *mgr*A gene (Figure 3). In addition to tetracycline, in our study, all strains were also resistant to penicillin and cefoxitin, and a high rate of resistance to gentamicin, tobramycin, trimethoprim–sulfamethoxazole, ciprofloxacin, and erythromycin was also detected. This does not come as a great surprise since, according to the European Medicines Agency (EMA) report on sales of veterinary antimicrobial agents, in Portugal, the most frequently sold antimicrobials were tetracyclines, followed by penicillins, macrolides, lincosamides, fluoroquinolones, and aminoglycosides [38]. Corroborating our results, Abreu et al. reported a notable rise in MRSA resistance among pigs to gentamicin, tobramycin, fosfomycin, clindamycin, trimethoprim–sulfamethoxazole, and tigecycline from 2009 to 2018 [39]. The resistance pattern observed in MRSA isolates from pigs in your study reflects widespread concerns regarding the proliferation and adaptation of antimicrobial resistance within LA-MRSA strains. The documented resistance to penicillin, cefoxitin, tetracycline, and clindamycin aligns with the typical attributes of MRSA ST398, a strain commonly linked to livestock and recognized for its extensive drug resistance. The elevated resistance levels to gentamicin, tobramycin, trimethoprim–sulfamethoxazole, ciprofloxacin, and erythromycin underscore the remarkable adaptability and resilience of these bacteria in response to antimicrobial pressures. In our study, resistance to penicillins was encoded by *bla*Z and *mec*A. Most isolates are resistant to macrolides and lincosamides carrying the *erm*B, *erm*C, *erm*T, *vga*(A)_LC_ and/or *vga*(A)_V_. The expression of *erm*B, *erm*C, and *erm*T is inducible by erythromycin and confers macrolide–lincosamide–streptogramin B (MLSb) resistance phenotype to the isolates [40,41]. Interestingly, four isolates carried the *erm*T gene, which is very uncommon in MRSA CC398 strains [42]. Nevertheless, other studies have reported the presence of this gene in MRSA from pigs [42,43,44]. The *vga*(A) is the most prevalent among the various *vga* genes identified in staphylococci, and they encode an ABC-F protein responsible for conferring resistance by shielding the ribosome against lincosamides, streptogramin A, and pleuromutilin antibiotics [45]. In our study, two different variants of *vga*(A) were identified, namely, *vga*(A)_LC_ and *vga*(A)_V_. The substrate spectrum includes streptogramin A, lincosamides, and pleuromutilins [46]. The *vga*(A)_LC_ gene was originally documented on plasmids identified in *S. haemolyticus* isolates from both feline and human sources in China [47]. Conversely, the *vga*(A)_V_ gene was found to be present in several copies per *S. aureus* isolate, with certain copies located within the chromosomal DNA and others detected on plasmids exceeding 40 kb in size [46]. Previous reports have documented *vga*(A)_LC_ in MRSA from Portuguese pigs, while both *vga*(A)_V_ and *vga*(A)_LC_ have been found in MRSA from German pigs [21,48]. Other genes that code for biocide efflux pumps, such as *mep*A, *nor*A, and *lmr*S, were also detected in all of our MRSA isolates, which may be attributed to the widespread use of biocides in animal husbandry [49]. On the other hand, the finding that only a minority of isolates displayed resistance to linezolid and mupirocin indicates that, despite widespread multidrug resistance, certain treatment alternatives may retain efficacy against these strains, suggesting promising avenues for treatment options or decolonization strategies [50].

LA-MRSA strains, including those within CC398, typically lack virulence genes associated with severe human infections, such as the IEC genes, genes encoding toxic shock syndrome toxin, and Panton–Valentine leucocidin (PVL) [51]. Accordingly, in our study, the *tst* gene was not detected, as were PVL and IEC-encoding genes in MRSA isolates. It has been shown that CC398 strains are often linked to elevated levels of antimicrobial resistance genes, which contrasts with the relatively low detection of virulence genes [26,52]. However, our isolates carried a high number of virulence genes. All 12 genes coding for the capsular polysaccharide synthesis enzymes were detected in all isolates (*cap*8A-P). The capsular polysaccharide is responsible for forming a dense layer on the outermost surface of the cell envelope in many *S. aureus* strains. These strains typically possess either a *cap*5A-P or a *cap*8A-P gene cluster, which codes for enzymes associated with the production of two common capsular serotypes, CP5 and CP8, respectively [53,54]. Evidence suggests that the advantageous role of capsule presence or absence in vivo varies depending on the infection context for *S. aureus* pathogenicity [55,56]. Other genes, such as *clf*A, *sdr*E, and *ebps*, were detected in all isolates. These genes are known to encode for the “microbial surface components recognizing adhesive matrix molecules” (MSCRAMMs). Many MSCRAMMs are pivotal not only in facilitating attachment and biofilm formation but also in invading host cells and tissues, evading immune responses, and bolstering virulence [57,58]. By binding to various host extracellular matrix proteins like fibronectin, fibrinogen, laminin, and elastin, along with indwelling medical devices and plasma-coated biological surfaces, these proteins aid in the initial attachment to native tissues [59]. In staphylococcal biofilms, numerous polysaccharides have been identified. However, the foremost factor governing the attachment and proliferation stages is the polysaccharide intercellular adhesion (PIA). PIA production relies on four genes, namely *ica*ADBC, which constitute the *ica* operon [60]. In our study, all isolates harbored the four *ica* genes (*ica*A, *ica*B, *ica*C, and *ica*D). Concerning the proteins of the type VII secretion system (T7), the *S. aureus* type VII protein secretion system (T7SS) is encoded within the *ess* locus. All isolates examined contained the corresponding coding genes, recognized as highly conserved core components of the T7 secretion apparatus [61]. In *S. aureus*, the *isd* genes are activated in response to iron scarcity, with their promoters regulated by the ferric uptake regulator. This system consists of nine proteins (IsdA-IsdI) primarily tasked with hemoglobin binding and heme extraction, ultimately transporting heme into the cytoplasm to serve as a source of iron [62]. Seven of the nine genes encoding for iron-regulated surface determinant proteins were detected in all isolates. The absence of available iron triggers the upregulation of several genes, including those encoding surface determinant (Isd) proteins. The main function of Isd proteins is to capture heme from hemoglobin (Hb) and facilitate its transportation into the cell [62,63]. Finally, as expected, the genes coding for hemolysins were identified in all MRSA isolates, which are very conserved genes that contribute to an invasive skin infection [64].

## 5. Conclusions

This study sheds light on the prevalence, antimicrobial resistance patterns, and molecular characteristics of MRSA among healthy swine populations in central Portugal. Our findings reveal a concerning prevalence of MRSA, particularly among fattening pigs and breeding sows, highlighting the potential role of pigs as reservoirs for MRSA transmission to humans. The predominance of the ST398 clonal complex, with diverse spa types identified, underscores the genetic diversity and epidemiological dynamics of MRSA in pig populations. Antimicrobial susceptibility testing revealed high levels of resistance to multiple antimicrobial agents, emphasizing the urgent need for prudent antimicrobial use practices in animal husbandry. Whole genome sequencing (WGS) analysis provided insights into the genetic determinants of antimicrobial resistance, with the detection of a diverse array of resistance genes. Additionally, the presence of virulence genes underscores the potential pathogenicity of MRSA strains and their ability to cause disease in both animals and humans.

Overall, continued surveillance and control measures are essential to monitor and mitigate the spread of MRSA in swine populations. Enhanced biosecurity measures, prudent antimicrobial use practices, and targeted intervention strategies are necessary to reduce the prevalence of MRSA in pigs and minimize the risk of zoonotic transmission to humans. Further research is warranted to elucidate the transmission dynamics and virulence potential of MRSA in swine populations, with the goal of safeguarding public health and ensuring food safety.

## Data Availability

Sequencing data generated within this study are deposited in Genbank under the BioProject ID PRJNA1006036.

## References

[1] Crespo-Piazuelo D., Lawlor P.G. (2021). Livestock-associated methicillin-resistant *Staphylococcus aureus* (LA-MRSA) prevalence in humans in close contact with animals and measures to reduce on-farm colonization. Ir. Vet. J..

[2] Cuny C., Wieler L., Witte W. (2015). Livestock-Associated MRSA: The Impact on Humans. Antibiotics.

[3] Pantosti A. (2012). Methicillin-resistant *Staphylococcus aureus* associated with animals and its relevance to human health. Front. Microbiol..

[4] Kayano T., Pulford J., Thomas L.F. (2023). Identifying Pig- and Pork-Associated Zoonotic and Foodborne Hazards in Eastern and Southern Africa: A Systematized Review. Zoonotic Dis..

[5] Price L.B., Stegger M., Hasman H., Aziz M., Larsen J., Andersen P.S., Pearson T., Waters A.E., Foster J.T., Schupp J. (2012). *Staphylococcus aureus* CC398: Host adaptation and emergence of methicillin resistance in livestock. MBio.

[6] van Wamel W.J.B., Rooijakkers S.H.M., Ruyken M., van Kessel K.P.M., van Strijp J.A.G. (2006). The innate immune modulators staphylococcal complement inhibitor and chemotaxis inhibitory protein of *Staphylococcus aureus* are located on β-hemolysin-converting bacteriophages. J. Bacteriol..

[7] Lakhundi S., Zhang K. (2018). Methicillin-Resistant *Staphylococcus aureus*: Molecular Characterization, Evolution, and Epidemiology. Clin. Microbiol. Rev..

[8] Food and Agriculture Organization (2022). World Agriculture: Towards 2015/2030.

[9] OECD, Food and Agriculture Organization of the United Nations (2021). OECD-FAO Agricultural Outlook 2021–2030.

[10] Abebe E., Gugsa G., Ahmed M. (2020). Review on Major Food-Borne Zoonotic Bacterial Pathogens. J. Trop. Med..

[11] Bidaisee S., Macpherson C.N.L. (2014). Zoonoses and one health: A review of the literature. J. Parasitol. Res..

[12] Heredia N., García S. (2018). Animals as sources of food-borne pathogens: A review. Anim. Nutr..

[13] Bellini S. (2021). 7. The pig sector in the European Union. Understanding and Combatting African Swine Fever.

[14] Rauw W.M., Rydhmer L., Kyriazakis I., Øverland M., Gilbert H., Dekkers J.C.M., Hermesch S., Bouquet A., Gómez Izquierdo E., Louveau I. (2020). Prospects for sustainability of pig production in relation to climate change and novel feed resources. J. Sci. Food Agric..

[15] Pirolo M., Visaggio D., Gioffrè A., Artuso I., Gherardi M., Pavia G., Samele P., Ciambrone L., Di Natale R., Spatari G. (2019). Unidirectional animal-to-human transmission of methicillin-resistant *Staphylococcus aureus* ST398 in pig farming; evidence from a surveillance study in southern Italy. Antimicrob. Resist. Infect. Control.

[16] Golob M., Pate M., Kušar D., Zajc U., Papić B., Ocepek M., Zdovc I., Avberšek J. (2022). Antimicrobial Resistance and Molecular Characterization of Methicillin-Resistant *Staphylococcus aureus* from Two Pig Farms: Longitudinal Study of LA-MRSA. Antibiotics.

[17] Moodley A., Nielsen S.S., Guardabassi L. (2011). Effects of tetracycline and zinc on selection of methicillin-resistant *Staphylococcus aureus* (MRSA) sequence type 398 in pigs. Vet. Microbiol..

[18] Mesa Varona O., Chaintarli K., Muller-Pebody B., Anjum M.F., Eckmanns T., Norström M., Boone I., Tenhagen B.-A. (2020). Monitoring antimicrobial resistance and drug usage in the human and livestock sector and foodborne antimicrobial resistance in six European countries. Infect. Drug Resist..

[19] European Food Safety Authority (EFSA), European Centre for Disease Prevention and Control (ECDC) (2023). The European Union Summary Report on Antimicrobial Resistance in zoonotic and indicator bacteria from humans, animals and food in 2020/2021. EFSA J..

[20] Conceição T., De Lencastre H., Aires-De-Sousa M. (2017). Frequent isolation of methicillin resistant *Staphylococcus aureus* (MRSA) ST398 among healthy pigs in Portugal. PLoS ONE.

[21] Leão C., Clemente L., Cara d’Anjo M., Albuquerque T., Amaro A. (2022). Emergence of Cfr-Mediated Linezolid Resistance among Livestock-Associated Methicillin-Resistant *Staphylococcus aureus* (LA-MRSA) from Healthy Pigs in Portugal. Antibiotics.

[22] Sasaki Y., Yamanaka M., Nara K., Tanaka S., Uema M., Asai T., Tamura Y. (2020). Isolation of ST398 methicillin-resistant *Staphylococcus aureus* from pigs at abattoirs in Tohoku region, Japan. J. Vet. Med. Sci..

[23] Sousa M., Silva N., Manageiro V., Ramos S., Coelho A., Gonçalves D., Caniça M., Torres C., Igrejas G., Poeta P. (2017). First report on MRSA CC398 recovered from wild boars in the north of Portugal. Are we facing a problem?. Sci. Total Environ..

[24] Porrero M.C., Mentaberre G., Sánchez S., Fernández-Llario P., Casas-Díaz E., Mateos A., Vidal D., Lavín S., Fernández-Garayzábal J.-F., Domínguez L. (2014). Carriage of *Staphylococcus aureus* by Free-Living Wild Animals in Spain. Appl. Environ. Microbiol..

[25] Quero S., Serras-Pujol M., Párraga-Niño N., Torres C., Navarro M., Vilamala A., Puigoriol E., de los Ríos J.D., Arqué E., Serra-Pladevall J. (2023). Methicillin-resistant and methicillin-sensitive *Staphylococcus aureus* in pork industry workers, Catalonia, Spain. One Health.

[26] Santos V., Gomes A., Ruiz-Ripa L., Mama O.M., Sabença C., Sousa M., Silva V., Sousa T., Vieira-Pinto M., Igrejas G. (2020). Methicillin-Resistant *Staphylococcus aureus* CC398 in Purulent Lesions of Piglets and Fattening Pigs in Portugal. Microb. Drug Resist..

[27] Kinross P., Petersen A., Skov R., Van Hauwermeiren E., Pantosti A., Laurent F., Voss A., Kluytmans J., Struelens M.J., Heuer O. (2017). Livestock-associated meticillin-resistant *Staphylococcus aureus* (MRSA) among human MRSA isolates, European Union/European Economic Area countries, 2013. Eurosurveillance.

[28] Silva V., Araújo S., Monteiro A., Eira J., Pereira J.E., Maltez L., Igrejas G., Lemsaddek T.S., Poeta P. (2023). *Staphylococcus aureus* and MRSA in Livestock: Antimicrobial Resistance and Genetic Lineages. Microorganisms.

[29] Wang Y., Zhang P., Wu J., Chen S., Jin Y., Long J., Duan G., Yang H. (2023). Transmission of livestock-associated methicillin-resistant *Staphylococcus aureus* between animals, environment, and humans in the farm. Environ. Sci. Pollut. Res..

[30] Albert E., Sipos R., Perreten V., Tóth Á., Ungvári E., Papp M., Dán Á., Biksi I. (2023). High Prevalence of Livestock-Associated Methicillin-Resistant *Staphylococcus aureus* in Hungarian Pig Farms and Genomic Evidence for the Spillover of the Pathogen to Humans. Transbound. Emerg. Dis..

[31] van der Mee-Marquet N., Corvaglia A.-R., Valentin A.-S., Hernandez D., Bertrand X., Girard M., Kluytmans J., Donnio P.-Y., Quentin R., François P. (2013). Analysis of prophages harbored by the human-adapted subpopulation of *Staphylococcus aureus* CC398. Infect. Genet. Evol..

[32] Cuny C., Abdelbary M., Layer F., Werner G., Witte W. (2015). Prevalence of the immune evasion gene cluster in *Staphylococcus aureus* CC398. Vet. Microbiol..

[33] Stegger M., Liu C.M., Larsen J., Soldanova K., Aziz M., Contente-Cuomo T., Petersen A., Vandendriessche S., Jiménez J.N., Mammina C. (2013). Rapid Differentiation between Livestock-Associated and Livestock-Independent *Staphylococcus aureus* CC398 Clades. PLoS ONE.

[34] Porrero M.C., Mentaberre G., Sánchez S., Fernández-Llario P., Gómez-Barrero S., Navarro-Gonzalez N., Serrano E., Casas-Díaz E., Marco I., Fernández-Garayzabal J.F. (2013). Methicillin resistant *Staphylococcus aureus* (MRSA) carriage in different free-living wild animal species in Spain. Vet. J..

[35] Pantosti A., Sanchini A., Monaco M. (2007). Mechanisms of antibiotic resistance in *Staphylococcus aureus*. Futur. Microbiol..

[36] Truong-Bolduc Q.C., Bolduc G.R., Medeiros H., Vyas J.M., Wang Y., Hooper D.C. (2015). Role of the Tet38 efflux pump in *Staphylococcus aureus* internalization and survival in epithelial cells. Infect. Immun..

[37] Truong-Bolduc Q.C., Dunman P.M., Strahilevitz J., Projan S.J., Hooper D.C. (2005). MgrA is a multiple regulator of two new efflux pumps in *Staphylococcus aureus*. J. Bacteriol..

[38] European Medicines Agency (2022). European Surveillance of Veterinary Antimicrobial Consumption, 2021. ‘Sales of Veterinary Antimicrobial Agents in 31 European Countries in 2019 and 2020’ EMA/58183/2021.

[39] Abreu R., Rodríguez-Álvarez C., Lecuona M., Castro B., González J.C., Aguirre-Jaime A., Arias Á. (2019). Increased antimicrobial resistance of MRSA strains isolated from pigs in Spain between 2009 and 2018. Vet. Sci..

[40] Li M., Cai C., Chen J., Cheng C., Cheng G., Hu X., Liu C. (2016). Inducible Expression of both *ermB* and *ermT* Conferred High Macrolide Resistance in *Streptococcus gallolyticus* subsp. pasteurianus Isolates in China. Int. J. Mol. Sci..

[41] Tkadlec J., Vařeková E., Pantůček R., Doškař J., Růžičková V., Botka T., Fila L., Melter O. (2015). Characterization of *Staphylococcus aureus* strains isolated from Czech cystic fibrosis patients: High rate of ribosomal mutation conferring resistance to MLSB antibiotics as a result of long-term and low-dose azithromycin treatment. Microb. Drug Resist..

[42] Abdullahi I.N., Lozano C., Zarazaga M., Simón C., Höfle U., Sieber R.N., Latorre-Fernández J., Stegger M., Torres C. (2024). Comparative genomics of *Staphylococcus aureus* strains from wild birds and pig farms elucidates levels of mobilomes, antibiotic pressure and host adaptation. J. Glob. Antimicrob. Resist..

[43] Mama O.M., Morales L., Ruiz-Ripa L., Zarazaga M., Torres C. (2020). High prevalence of multidrug resistant *S. aureus*-CC398 and frequent detection of enterotoxin genes among non-CC398 *S. aureus* from pig-derived food in Spain. Int. J. Food Microbiol..

[44] Abdullahi I.N., Lozano C., Simon C., Latorre-Fernandez J., Zarazaga M., Torres C. (2023). Nasal staphylococci community of healthy pigs and pig-farmers in Aragon (Spain). Predominance and within-host resistome diversity in MRSA-CC398 and MSSA-CC9 lineages. One Health.

[45] Feßler A., Kadlec K., Wang Y., Zhang W.-J., Wu C., Shen J., Schwarz S. (2018). Small antimicrobial resistance plasmids in livestock-associated methicillin-resistant *Staphylococcus aureus* CC398. Front. Microbiol..

[46] Feßler A.T., Wang Y., Wu C., Schwarz S. (2018). Mobile lincosamide resistance genes in staphylococci. Plasmid.

[47] Deng F., Wang H., Liao Y., Li J., Feßler A.T., Michael G.B., Schwarz S., Wang Y. (2017). Detection and genetic environment of pleuromutilin-lincosamide-streptogramin A resistance genes in staphylococci isolated from pets. Front. Microbiol..

[48] Henrike K.-H., Xing J., Dennis H., Stefan F., Feßler A.T., Nansong J., Heike K., Yang W., Congming W., Stefan S. (2023). Genomic Diversity of Methicillin-Resistant *Staphylococcus aureus* CC398 Isolates Collected from Diseased Swine in the German National Resistance Monitoring Program GERM-Vet from 2007 to 2019. Microbiol. Spectr..

[49] Soliman E.S., Moawed S.A., Ziaan A.M.G. (2016). Assessing cleaning and disinfection regime in a slaughterhouse against carcasses contamination. Adv. Anim. Vet. Sci.

[50] Asanin J., Misic D., Aksentijevic K., Tambur Z., Rakonjac B., Kovacevic I., Spergser J., Loncaric I. (2019). Genetic Profiling and Comparison of Human and Animal Methicillin-Resistant *Staphylococcus aureus* (MRSA) Isolates from Serbia. Antibiotics.

[51] Silva V., Vieira-Pinto M., Saraiva C., Manageiro V., Reis L., Ferreira E., Caniça M., Capelo J.L., Igrejas G., Poeta P. (2021). Prevalence and Characteristics of Multidrug-Resistant Livestock-Associated Methicillin-Resistant *Staphylococcus aureus* (LA-MRSA) CC398 Isolated from Quails (*Coturnix Coturnix Japonica*) Slaughtered for Human Consumption. Animals.

[52] Argudín M.A., Tenhagen B.A., Fetsch A., Sachsenröder J., Käsbohrer A., Schroeter A., Hammer J.A., Hertwig S., Helmuth R., Bräunig J. (2011). Virulence and Resistance Determinants of German *Staphylococcus aureus* ST398 Isolates from Nonhuman Sources. Appl. Environ. Microbiol..

[53] Weidenmaier C., Lee J.C. (2017). Structure and function of surface polysaccharides of *Staphylococcus aureus*. Staphylococcus aureus Microbiology, Pathology, Immunology, Therapy and Prophylaxis.

[54] Keinhoerster D., George S.E., Weidenmaier C., Wolz C. (2019). Function and regulation of *Staphylococcus aureus* wall teichoic acids and capsular polysaccharides. Int. J. Med. Microbiol..

[55] O’Riordan K., Lee J.C. (2004). *Staphylococcus aureus* capsular polysaccharides. Clin. Microbiol. Rev..

[56] Tuchscherr L., Löffler B., Buzzola F.R., Sordelli D.O. (2010). *Staphylococcus aureus* adaptation to the host and persistence: Role of loss of capsular polysaccharide expression. Future Microbiol..

[57] Kang M., Ko Y.-P., Liang X., Ross C.L., Liu Q., Murray B.E., Höök M. (2013). Collagen-binding microbial surface components recognizing adhesive matrix molecule (MSCRAMM) of Gram-positive bacteria inhibit complement activation via the classical pathway. J. Biol. Chem..

[58] Foster T.J. (2019). The MSCRAMM Family of Cell-Wall-Anchored Surface Proteins of Gram-Positive Cocci. Trends Microbiol..

[59] Silva V., Capelo J.L., Igrejas G., Poeta P., Akhtar N., Singh K.S., Prerna, Goyal D. (2022). Molecular Mechanisms of Antimicrobial Resistance in Staphylococcus aureus Biofilms BT—Emerging Modalities in Mitigation of Antimicrobial Resistance.

[60] Miao J., Lin S., Soteyome T., Peters B.M., Li Y., Chen H., Su J., Li L., Li B., Xu Z. (2019). Biofilm Formation of *Staphylococcus aureus* under Food Heat Processing Conditions: First Report on CML Production within Biofilm. Sci. Rep..

[61] Bowman L., Palmer T. (2021). The type VII secretion system of *Staphylococcus*. Annu. Rev. Microbiol..

[62] Zapotoczna M., Heilbronner S., Speziale P., Foster T.J. (2012). Iron-regulated surface determinant (Isd) proteins of *Staphylococcus lugdunensis*. J. Bacteriol..

[63] Pietrocola G., Pellegrini A., Alfeo M.J., Marchese L., Foster T.J., Speziale P. (2020). The iron-regulated surface determinant B (IsdB) protein from *Staphylococcus aureus* acts as a receptor for the host protein vitronectin. J. Biol. Chem..

[64] Pineda A.P.A., Cueva C.L.R., Chacón R.D., Ramírez M., De Almeida O.G.G., De Oliveira D.P., Franco B.D.G.M., Lacorte G., Landgraf M., Silva N.C.C. (2023). Genomic characterization of *Staphylococcus aureus* from Canastra Minas Artisanal Cheeses. Braz. J. Microbiol..

